# Phosphorus in Locomotor Ataxia

**Published:** 1869-04

**Authors:** Walter Lambert

**Affiliations:** Amherstburg, Ontario, Canada


### PHOSPHORUS IN LOCOMOTOR ATAXIA.

By WALTER LAMBERT, M.B., Amherstburg, Ontario, Canada.

Miss F. B., aged 22, had been suffering slightly with anæmia and scanty menstruation for about one year. At different times, she took ferruginous preparations, with decidedly good effects; but, as soon as relieved, she would leave off taking the medicine, and her trouble would return. She also had ague once or twice during the summer, it being very prevalent at that time in the neighborhood. For it she was specifically treated, and from it she soon recovered.

For the chlorosis I sometimes gave mistura ferri comp. (Griffith's), sometimes tinct. ferri and quiniæ disulph.; lastly, I was giving her syr. ferri iodidi, with cod-liver oil. In September last, from exposure to wet and cold, her menses ceased, and all the symptoms of progressive locomotor ataxia set in. Her parents, who live in the country, came for more medicine, and casually told me that their daughter walked with great difficulty, and that her menses did not come on at their usual period; consequently, I went to see her, and, in her attempting to shake hands with me, she grasped me by the wrist. This excited my fears immediately that she had Duchenne's disease. Upon further examination, my diagnosis was verified. The patient, in attempting to walk, staggered and swayed her body from side to side to keep her equilibrium. She would suddenly halt to recover herself, and then would plunge forward, seemingly in a great hurry to reach the point to which she desired to go. She was unable to feed herself, from the want of coördinate action of the muscles; and, in fact, unless she was watching her hands continually, she was liable to drop whatever she had in them. Her speech was also affected; she was not able to articulate some words perfectly.

What is passing strange in this case is, that I was giving her syr. ferri iodidi at the very time that the disease manifested itself; the very medicine that Dr. Julius Althaus used with so much benefit in his case, the only one recorded, until lately, that had been much benefited by medicine.

As soon as I recognized the disease, I gave potass. bromid. grs. xv., ter in die, and submitted the patient to the action of magneto-electricity once every twenty-four hours. I also gave two pills of aloes and iron, which produced too much relaxation, the effect continuing two or three days. This, in fact, seemed to prostrate her to such an extent that she was obliged to take to her bed, and there remain for a time. Fortunately, just then I received the September number of the *New York Medical Journal*, and in it saw that Dr. Dujardin Baumetz had given phosphorus in this disease, with excellent effects. I immediately ordered acidi phosphorici dilut. m. xv., ter in die, in simple syrup. The next day her menses came on, and in a short time she began to improve. In a few days I increased the dose to twenty, twenty-five, and then to thirty minims.

After ten or twelve days, I omitted the acid, and gave her the pyro-phosphate of iron for a week, and then returned to the acid. I continued the electricity every alternate day. In two weeks she was able to sit up, and had sufficient control over the muscles of her upper extremities to be able to knit. In one month she could walk about the house tolerably well. Now it is something over two months; she can take long walks, do housework as well as ever, and has become very fleshy. The electricity has been discontinued for about one month, and she is not at all regular with her medicine at the present time. However, I have the most sanguine hopes that she will perfectly recover. The improvement has been so great that it is impossible to discern anything wrong with her, except a very slight irregularity in her walk.*—*New York Medical Journal.*

* We are informed by Dr. Lambert, in a note received since this article was in type, that the patient has perfectly recovered under this treatment.—E. S. D.

